# Linking the neural signature of response time variability to Alzheimer’s disease pathology and cognitive functioning

**DOI:** 10.1162/netn_a_00373

**Published:** 2024-10-01

**Authors:** James Teng, Michael R. McKenna, Oyetunde Gbadeyan, Ruchika S. Prakash, for the Alzheimer’s Disease Neuroimaging Initiative

**Affiliations:** Department of Psychology, The Ohio State University, Columbus, OH, USA; Center for Cognitive and Behavioral Brain Imaging, The Ohio State University, Columbus, OH, USA; National Centre for Healthy Ageing, Peninsula Clinical School, Faculty of Medicine, Monash University, Melbourne, Australia

**Keywords:** Mind-wandering, Response time variability, Alzheimer’s disease, Connectome-based predictive model, fMRI, Functional connectivity

## Abstract

Promising evidence has suggested potential links between mind-wandering and Alzheimer’s disease (AD). Yet, older adults with diagnosable neurocognitive disorders show reduced meta-awareness, thus questioning the validity of probe-assessed mind-wandering in older adults. In prior work, we employed response time variability as an objective, albeit indirect, marker of mind-wandering to identify patterns of functional connectivity that predicted mind-wandering. In the current study, we evaluated the association of this connectome-based, mind-wandering model with cerebral spinal fluid (CSF) p-tau/A*β*_42_ ratio in 289 older adults from the Alzheimer’s Disease NeuroImaging Initiative (ADNI). Moreover, we examined if this model was similarly associated with individual differences in composite measures of global cognition, episodic memory, and executive functioning. Edges from the high response time variability model were significantly associated with CSF p-tau/A*β* ratio. Furthermore, connectivity strength within edges associated with high response time variability was negatively associated with global cognition and episodic memory functioning. This study provides the first empirical support for a link between an objective neuromarker of mind-wandering and AD pathophysiology. Given the observed association between mind-wandering and cognitive functioning in older adults, interventions targeted at reducing mind-wandering, particularly before the onset of AD pathogenesis, may make a significant contribution to the prevention of AD-related cognitive decline.

## Author Summary

Response time variability is considered an objective, albeit indirect, marker of mind-wandering. In this study, we applied a previously-derived connectome-based model of response time variability to resting-state data obtained from 289 older adults in the Alzheimer’s Disease NeuroImaging Initiative. The network strength of the high response time variability model was correlated with a cerebrospinal fluid (CSF)-based ratiometric measure of amyloid and tau pathology. Additionally, our results demonstrated that the network strength in the high response time variability model was also linked with global cognition and episodic memory. This study provides the first empirical support for the association between a neuromarker of response time variability—an indirect marker of mind-wandering—and AD pathophysiology.

## INTRODUCTION

Mind-wandering is considered a common human phenomenon with adults endorsing such experiences in 30%–50% of their waking times. Traditionally defined as the occurrence of stimulus-independent thoughts during an externally oriented task (Smallwood & Schooler, 2006), mind-wandering has been quantified using thought probes embedded in tasks of sustained attention (Giambra, 1989). These self-reported probes are designed to inquire about the content and nature of thought processes right before the presentation of the probe, and though there has been considerable heterogeneity in the literature on the structuring and wording of these thought probes (see Seli et al., 2018, for a discussion on this topic), there is an emerging consensus that mind-wandering is a multidimensional construct that captures a range of experiences (Groot et al., 2021; Kane et al., 2007).

A more recent neural model of mind-wandering also postulates that, rather than truly reflecting mind-wandering, these self-reported thought probes capture an intermediate off-focus, or exploratory state that lies between on-task thinking and mind-wandering (Mittner et al., 2016). This “off-focus,” exploratory state that follows the state of sustained attention is characterized by increased functional connectivity across many canonical networks of the brain. One of the key features that distinguishes the off-focus state from the mind-wandering state is the impact on behavioral performance as off-focus exploration is associated with modest impact on behavioral performance, whereas the state of mind-wandering is characterized by significant variability in behavioral performance. According to this model then, the reaction time coefficient of variation, the trial-to-trial fluctuation in reaction time, is a better, albeit indirect, indicator of the mind-wandering state. Supporting this conjecture, increased individual variability in reaction time has, indeed, been associated with self-reports of mind-wandering episodes (Bastian & Sackur, 2013; Henríquez et al., 2016; Jubera-García et al., 2020; Kucyi et al., 2016; Maillet et al., 2020) as well as other lapses in attention (Schooler et al., 2014). Moreover, response time variability has also been found to be higher on trials preceding off-task thought probes compared to on-task probes (Seli et al., 2013).

Interestingly, the aging literature provides support for the differential trajectories of metrics of mind-wandering with increasing age. Self-reported mind-wandering, assessed through thought probes, and capturing the ability to direct resources to off-task thinking amid cognitively taxing tasks, tends to decline with age (Jackson & Balota, 2012)—including in individuals with mild cognitive impairment and AD (Niedźwieńska & Kvavilashvili, 2018; O’Callaghan et al., 2019). Though there are theoretical models that explain lower endorsement of mind-wandering probes as reflective of fewer available cognitive resources to engage in mind-wandering in older adults (Smallwood & Schooler, 2006), others have provided evidence for reduced meta-awareness with advancing age, particularly in those with neurocognitive disorders (Rosen et al., 2014). In contrast, response time variability follows the hypothesized association with age as a more objective marker of mind-wandering. Older adults demonstrate higher response time variability compared with young adults (Zavagnin et al., 2014), and high variability has robust consequences for cognitive functioning (Jackson et al., 2012).

Moreover, mind-wandering episodes are associated with reduced connectivity between the temporal and prefrontal regions of the default mode network (Martinon et al., 2019; O’Callaghan et al., 2015). In older adults, mind-wandering is correlated with a reduced engagement of the medial prefontal cortices, the lateral prefrontal cortices, and the left superior temporal gyrus (Maillet et al., 2019). Examining the neural correlates of mind-wandering in individuals with dementia, O’Callaghan and colleagues (2019) employed a minimally demanding Shapes Expectation Task. Using thought probe data, they computed a mind-wandering index to examine associations between mind-wandering, functional connectivity, and gray matter volume. In older adults with AD, the mind-wandering index was associated with reduced coupling of the posterior cingulate cortex (a metabolic hub of the default mode network), the hippocampus, and the prefrontal cortex. In a recent study, we leveraged connectome-based predictive modeling—a whole-brain and data-driven technique that allows for the derivation of brain-based predictive models from individualized functional connectivity patterns—to develop a neural model for response time variability (RT_CV CPM) in a cohort of 145 older adults, aged 65 to 85 years (Gbadeyan et al., 2022; Shen et al., 2017). Using data from the Human Connectome Project in Aging (Bookheimer et al., 2019), we identified functional connections during the Go/No-Go task that were predictive of high response time variability and functional connections that were predictive of low response time variability. The task-based predictive model was robust to the effects of age, sex, study sites, and the cross-validation method. Neuroanatomically, the whole-brain model provided support for the differential involvement of key canonical networks, including the default mode network, the somatomotor network, the dorsal attention network, the ventral attention network, the visual network, and the fronto-parietal network.

In this study, we extend the application of our task-based RT_CV CPM to more trait-like AD pathophysiology by investigating whether network strength in the high and low response time variability models is associated with a well-established cerebrospinal fluid-based marker of AD pathophysiology (p-tau/A*β*_42_ ratio) in resting-state fMRI. In prior work in our lab, we have shown that the combined ratiometric measure of amyloid and tau pathology (p-tau/A*β*_42_), was better at determining diagnostic status—cognitively normal, MCI, and AD—than either p-tau or A*β*_42_ alone (Prakash et al., 2020, Preprint). Thus, in the current study, we selected the CSF-based ratio of p-tau/A*β*_42_ as a metric for AD pathophysiology. Employing neuroimaging and cerebrospinal fluid-based data available dataset from the Alzheimer’s Disease Neuroimaging Initiative (Mueller et al., 2005), we computed network strength in the high and low mind-wandering models. We hypothesized that network strength in the high RT_CV model would be associated with higher levels of p-tau/A*β*_42_, suggesting that high response time variability is linked with greater levels of AD pathophysiology. For the low RT_CV model, we hypothesized that network strength would be negatively associated with pathophysiology levels. And, finally, to directly examine the functional significance of the response time variability models for cognitive performance, we also examined associations between network strengths in the high and low RT_CV models with cognitive functioning in the composites of global cognition, episodic memory, and executive functioning. To our knowledge, this is the first study to directly examine the functional edges involved in a response time variability connectome with that of fluid-based biomarkers to explore the shared connectomics between mind-wandering and AD pathophysiology.

## MATERIALS AND METHODS

### Data Overview

We analyzed the publicly available fMRI, cerebrospinal fluid biomarker, and behavioral data of 324 older adults aged 55–90 from the Alzheimer’s Disease Neuroimaging Initiative (ADNI; Petersen et al., 2010). In addition, the RT_CV models (Gbadeyan et al., 2022) utilized in this report was previously generated using data from the Human Connectome Project in Aging (HCP-Aging; Bookheimer et al., 2019).

### Participants

ADNI is an ongoing, multicenter study that has sought to define Alzheimer’s disease progression using a variety of modalities (PET, MRI, and cerebrospinal fluid-based biological markers, and a variety of neuropsychological assessments; see https://adni.loni.usc.edu/) as predictors of the disease. We used data from the three phases released thus far: ADNI-GO, ADNI-2, and ADNI-3. Data reported in the current manuscript were collected from 43 sites across the United States and Canada. The MRI, cognitive batteries, and lumbar punctures were collected across one and three study sessions. The MRI session and cognitive batteries were separated by an average of 7.69 days (*SD* = 15.5 days), the MRI and CSF measures were separated by an average of 7.38 days (*SD* = 33.5 days), and the cognitive batteries and CSF measures were separated by an average of 13.23 days (*SD* = 26.6 days). Per ADNI protocols, efforts were made to minimize intersite differences through the use of standardized data collection protocols (Jack et al., 2008, 2015; Weber et al., 2021). To our knowledge, there have been little systematic differences in protocols across the various sites (Nir et al., 2013), and thus, data harmonization was not commonly performed across ADNI studies (Jack et al., 2008, 2015; Weber et al., 2021).

Healthy participants were between 55 and 90 years old at time of recruitment, were fluent in either English or Spanish, and scored less than six on the Geriatric Depression Scale. A total of 324 participants were selected across all phases of ADNI. Of these 324 individuals, participants were removed due to poor brain coverage or global signal in their fMRI data (*n* = 5), and those with excessive head motion (*n* = 30) during the resting-state fMRI scan (mean framewise displacement > .15 mm) were excluded from subsequent analyses. In sum, data from 289 participants were used for all analyses in this report. Of these, the cognitively normal group comprised of 149 individuals (89 females, mean age (*SD*) = 72.6 (7.00)), the MCI group comprised of 109 individuals (48 females, mean age (*SD*) = 71.5 (7.30)), and the AD group comprised 31 individuals (13 females, mean age (*SD*) = 73.5 (7.92)). For tests employed to determine diagnostic status, please see the Supporting Information.

### Neuropsychological Assessments and Cerebrospinal Fluid-Based Biomarkers

Participants in the ADNI study were administered a large battery of neuropsychological tests to examine a variety of cognitive domains, including global cognition, episodic memory, executive function, spatial orientation, processing speed, and language. Pertinent to this report, we chose preexisting, validated assessments that were available in cognitive domains commonly implicated in AD (Donohue et al., 2014): global cognition, episodic memory, and executive function.

#### Cognitive composites.

The Preclinical Alzheimer’s Cognitive Composite (PACC) characterized global cognitive deficits in preclinical AD, and includes the following measures: the Mini-Mental Status Examination total score, the Trails-Making Test B score, the delayed recall score from the Logical Memory II subscale, and the delayed word recall from the Alzheimer’s Disease Assessment Scale Cognitive Subscale (ADAS-COG). To index episodic memory, we employed the ADNI-Mem composite. This summary measure included performance on the Logical Memory I and II tasks, several item scores on the Rey Auditory Verbal Learning Test, the cognitive subscale of the Alzheimer’s Disease Assessment Scale, and the three word recall items from the Mini-Mental State Examination. Finally, to index executive functioning, the ADNI-EF composite was employed, which included the Digit Symbol Substitution test from the Weschler Adult Intelligence Scale-Revised, the Digit Span Backwards Test, Trails-Making A and B, Category Fluency, and Clock Drawing. Baseline PACC scores were available in the adnimerge.rdata file, while baseline ADNI-MEM and ADNI-EF were extracted from the uwnpsychsum.rdata file nested in the ADNIMERGE R package.

#### Cerebrospinal fluid biomarkers.

The cerebrospinal fluid-based protein biomarkers were analyzed as the ratio of p-tau/A*β*_42_ (pg/mL) in the cerebrospinal fluid as measured by the automated Roche Elecsys immunoassays on the Cobas e601 system. As the primary assay of the current phase (ADNI3), the Roche Elecsys immunoassay was determined to provide better compatibility for potential future ADNI releases compared to the traditional AlzBio3 immunoassay. Of note, the measurement bounds of the Elecsys-based assay meant that while A*β*_42_ concentrations (200–1,700 pg/mL) were not extrapolated at the lower limits, extrapolation was performed on values at the upper limit via calibration curves by the ADNI group. We then computed a ratiometric measure of p-tau/A*β*_42_, with larger values indicating greater proteinopathy.

#### MRI processing and application of the connectome-based predictive modeling approach.

Details on the standardized structural and functional MRI data acquisition for the ADNI study are reported elsewhere (Jack et al., 2008, 2015) and summarized in the Supporting Information. Additionally, standard preprocessing pipelines were implemented on resting-state data and explained in detail in the Supporting Information. Postprocessed, whole-brain functional MRI data was parcellated into 268 contiguous, functionally defined regions (i.e., nodes) that covered the cortex, the subcortex, and the cerebellum (Shen et al., 2013). This functional atlas in MNI space was transformed into each participant’s native functional space to generate participant specific atlases, and the BOLD signal time course was extracted from each node. Six nodes were missing from three or more participants, and they were subsequently removed from all participants during analysis. Functional connectivity was then calculated as the Fisher’s z-transformed Pearson’s correlation coefficient between every possible node pair. The resulting 262 × 262 functional connectivity matrix represented the magnitude of the connection between every node (i.e., edges).

In this study, we were interested in examining whether network strength of the RT_CV CPM, originally derived in Gbadeyan et al. (2022), was associated with AD pathophysiology and cognitive functioning. The RT_CV masks in the original study were derived using connectome-based predictive modeling—a supervised machine learning algorithm designed to derive brain-based predictive models from individualized functional connectivity patterns. In the Gbadeyan et al. (2022) study, using a leave-one-out cross-validation approach, edges with the strongest positive correlations with response time variability (RT_CV) were selected for inclusion in the high RT_CV model (i.e., most positively correlated edges). In contrast, functional connections with the strongest negative correlations were included in the low response variability model (i.e., most negatively correlated edges). Subsequently, a linear model was fitted for each of the high and low response variability networks to generate predicted RT_CV from the left-out participant. The final high and low response variability masks included edges that occurred across each iteration of the leave-one-out cross-validation, resulting in a mask representing functional connections that were consistently associated positively with RT_CV and functional edges that were consistently associated negatively with RT_CV.

These final consensus masks of the high and low response variability models (262 × 262 symmetrical, binary matrices with 1 s for edges in the networks and 0 s elsewhere), were applied to the 289 participants’ functional connectivity matrices from the ADNI dataset to compute mean network summary strength scores. This resulted in a network strength score for the high RT_CV model and one network strength score for the low RT_CV model. The mean framewise displacement for participants in these analyses was low (FD mean = 0.0768 mm, *SD* = 0.0278). However, as head motion can be a significant confound in functional connectivity-based analyses, we examined associations between motion and network strengths in the high RT_CV CPM and the low RT_CV CPM. Motion was significantly associated with network strength in the high RT_CV model (*r* = .48, *p* < .0001) and the low RT_CV CPM strength (*r* = −0.22, *p* < .001). Thus, mean framewise displacement was included as a covariate in the subsequent analyses. Of note, CSF p-tau/A*β*_42_ ratio as well as all cognitive composites exhibited a nonnormal distribution in the current sample. As a result, Spearman’s correlations were employed to examine the associations between network strength in the high and low RT_CV models and AD pathophysiology and cognitive functioning, after controlling for the effects of motion.

Given the contributions of specific canonical networks to a neural signature of mind-wandering, we elected to test whether the association between network strength of the RT_CV CPM and AD metrics was limited to the functional connectivity of these key canonical networks. The relationships between mind-wandering and specific brain networks, including the default mode network (Fox et al., 2015), the dorsal attention network (Christoff et al., 2016), and the fronto-parietal network (O’Callaghan et al., 2019) have been well-established in prior research. As such, these networks were chosen as target regions for the application of computational lesioning in our study. Considering our previous research (Gbadeyan et al., 2022), the ventral attention network was also included given its overrepresentation in our RT_CV CPM. Edges from each of the four canonical networks—within network connections and any between-network connections—were excluded from participant functional connectivity matrices as well as consensus masks of the high, and low RT_CV CPM. For example, the computational lesioning of the default mode network resulted in the removal of all edges from within the 35 default mode network nodes, including both within- and between-network edges. RT_CV CPM was then applied to the remaining 228 × 228 functional connectivity matrices. We computed the correlation between network strength in the lesioned model and Alzheimer’s disease pathophysiology and cognitive functioning. Finally, differences in the associations between the whole-brain and lesioned models were tested using Steiger’s *Z* (Steiger, 1980).

## RESULTS

**Figure 1.**
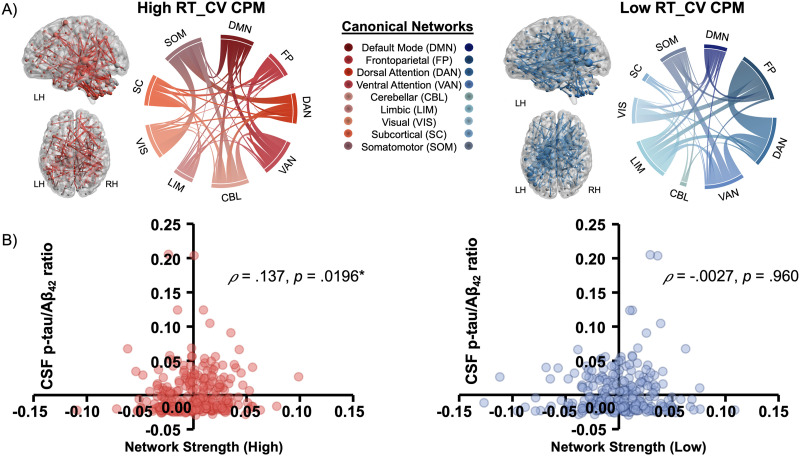
The anatomical distribution of predictive edges in the RT_CV CPM and the associations between network strength and AD pathology. (A) Predictive edges for the high (134 edges, in red) and low (134 edges, in blue) RT_CV CPMs. Predictive edges were further collapsed to their canonical networks and are visualized using chord diagrams. (B) Scatterplot of the Spearman’s correlations between summary network strength scores and cerebrospinal fluid-measured p-tau/A*β*_42_ levels for the high RT_CV CPM (in red) and the low RT_CV CPM (in blue).

A total of 289 participants from the ADNI database were included in this report (see Table 1 for the participant demographics and clinical characteristics). We evaluated the association between response time variability as an indirect marker of mind-wandering and AD pathophysiology by utilizing a previously established whole-brain functional connectivity-based neural signature of response time variability (Gbadeyan et al., 2022). Networks of the RT_CV CPM contained 134 edges in the high and low models, such that the high and low network included edges that were positively and negatively associated with response variability-based mind-wandering (Figure 1A). Importantly, in this study, we extended the model’s generalizability to a completely novel context—assessing the associations between network strength in the high and low RT_CV CPMs with cerebrospinal fluid biomarker levels in an independent group of participants.

**Table 1.** Baseline characteristics of participants

Characteristic		*n* = 289		
		Mean (*SD*) or *N* (%)		Range
Sex				
	Female	150	(51.9%)	
	Male	139	(48.1%)	
Race*				
	Asian	7	(2.4%)	
	Black	8	(2.8%)	
	More than one race	7	(2.4%)	
	White	266	(92%)	

Age (years)		72.3	(7.22)	55.5 to 91.5
Years of education		16.6	(2.32)	11 to 20
Diagnostic Status**			*ε*4 allele absent	*ε*4 allele(s) present
	CN	149	98 (66.2%)	50 (33.8%)
	MCI	109	60 (55%)	49 (45%)
	AD	31	5 (16%)	26 (84%)

*Note*. CN = cognitively normal; MCI = mild cognitive impairment; AD = Alzheimer’s disease.

* Race missing for one participant in the MCI group.

** *APOE* information missing for one participant in the CN group.

**Figure 2.**
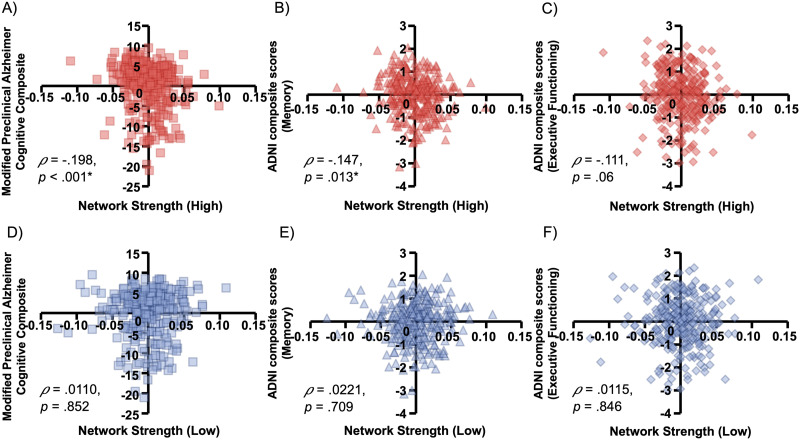
Associations between the high RT_CV CPM connectivity strength and cognitive composite scores. Scatterplots show the correlation between model-based connectivity strength from the high response time variability connectome-based predictive model and the observed scores from the (A) Preclinical Alzheimer’s Cognitive Composite (PACC), (B) ADNI-Memory (ADNI-Mem) composite, and (C) ADNI-Executive Functioning (ADNI-EF) composite. Correlations with the low response time variability connectome-based predictive model are also shown for the (D) PACC, (E) ADNI-MEM, and (F) ADNI-EF. Annotations represent Spearman’s correlation coefficients and *p* values.

We found that network strength in the high RT_CV CPM was significantly associated with observed p-tau/A*β*_42_ ratio after accounting for head motion (high model: *ρ* = .137, *p* = .0196). However, association with edges from the low RT_CV CPM was not significant (low model: *ρ* = −.0027, *p* = .960; see Figure 1B). We next examined the association between network strengths of the RT_CV CPM and cognitive functioning in the domains of general cognition, episodic memory, and executive functioning. Network strengths within the consensus mask of the high RT_CV CPM—functional edges that were associated with high behavioral variability across all participants—were negatively associated with global cognitive deficits and episodic memory (PACC: *ρ* = −.198, *p* < .001; ADNI-Mem: *ρ* = −.147, *p* = .013), but not executive function (ADNI-EF: *ρ* = −.111, *p* = .060; see Figure 2A–C). However, the low RT_CV CPM did not significantly correlate with the cognition composites (Figure 2D–F).

**Figure 3.**
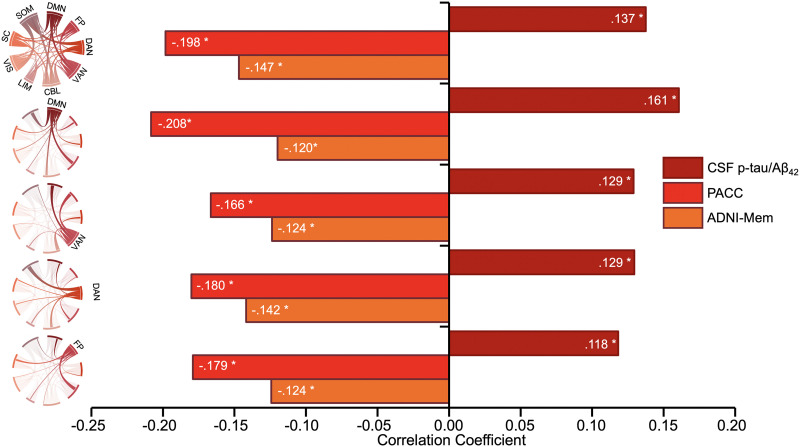
Network strength associations following computational lesion analyses. Bar graphs show the Spearman’s correlations between summary network strengths in the high RT_CV model and p-tau/A*β*_42_ levels, PACC, and ADNI-MEM before (top) and after lesioning the default mode network, ventral attention network, dorsal attention network, and fronto-parietal network, respectively.

Given that only the high RT_CV CPM was significantly associated with AD pathophysiology, global cognition, and memory functioning, we performed the computational lesion analyses only for these models. Results consistently showed that the model remained significantly associated with AD pathophysiology following the removal of nodes in the default mode network (*ρ* = .161, *p* = .0063), the ventral attention network (*ρ* = .129, *p* = .029), the dorsal attention network (*ρ* = .129, *p* = .028), and the fronto-parietal network (*ρ* = .118, *p* = .045), respectively. We found no significant differences between the association of whole-brain network strength and lesioned models’ network strength for CSF p-tau/A*β*_42_ (DMN: Steiger’s *Z* = −0.744, *p* = .457; VAN: Steiger’s *Z* = 0.422, *p* = .673; DAN: Steiger’s *Z* = 0.446, *p* = .656; FPN: Steiger’s *Z* = 1.18, *p* = .237). Similarly, network strength in the high RT_CV CPM was associated with global cognition and memory functioning even after the removal of nodes in each of these canonical networks (see Figure 3). None of the Steiger’s *Z* comparisons were statistically significant for global cognition or episodic memory.

## DISCUSSION

The primary goal of this study was to examine the association between a whole-brain, connectivity-based signature of mind-wandering (RT_CV CPM; Gbadeyan et al., 2022) and the proteinopathies of amyloid beta and tau pathology. We showed that network strength of the high mind-wandering model was positively associated with cerebrospinal fluid p-tau/AB_42_ ratio in an independent sample of mixed healthy, MCI, and AD participants. Although interest in the relationship between mind-wandering and AD has begun to gain traction in the field (Gyurkovics et al., 2018; Kvavilashvili et al., 2020; O’Callaghan et al., 2019), our findings here are the first to bridge the gap between a neural correlate of mind-wandering and AD pathophysiology. Additionally, consistent with our initial hypothesis, network strength in the high RT_CV CPM also had significant associations with cognitive domains that commonly show declines in AD, such as general cognition (Donohue et al., 2014) and memory (Kelley & Petersen, 2007). Although cross-sectional, our study results provide the first evidence for a direct link between functional connectivity patterns that predict response time variability—an indirect, yet objective marker of mind-wandering—and AD pathogenesis and cognitive functioning.

As hypothesized, edges within the high response time variability model were significantly associated with cerebrospinal fluid p-tau/A*β*_42_ levels from an independent, mixed pathology sample. These results suggest that older adults showing greater functional connectivity between nodes of this network also have high baseline levels of amyloid and tau pathology. Our results are consistent with the literature examining response time variability as a marker of decline in older adults with and without AD pathophysiology (Gorus et al., 2008). Across studies, older adults, including older adults with mild cognitive impairment and AD, show an increase in response time variability, suggesting that performance on cognitive tasks is more variable in older adults on the spectrum of pathological aging. Although mind-wandering has traditionally been investigated through the lens of self-caught probes, there is emerging consensus on the multidimensional nature of mind-wandering (Wang, Poerio, et al., 2018). Response time coefficient of variability—indexing the trial-to-trial fluctuations in reaction time—is considered an indirect, yet objective marker of mind-wandering (Seli et al., 2013). Furthermore, response time variability may indeed also capture the more goal-oriented state of mind-wandering, as opposed to the more exploratory, off-focus state captured through thought probes (Mittner et al., 2016), thus suggesting that the neural connections associated with high variation in response time has critical significance for understanding the neurobiological basis of mind-wandering. Extending this to the domain of AD pathophysiology, we showed that there may exist a closer association between AD pathophysiology and the neural signatures of mind-wandering than previously believed.

Furthermore, the edges critical to this network, primarily located in the subcortical, visual, and ventral attention networks (see Gbadeyan et al., 2022), represent a widespread distribution across multiple functional networks. Between-network contributions from the default mode network and the networks such as the ventral attention and fronto-parietal networks were also highly represented in the high RT_CV CPM. The functional neuroanatomy of our high RT_CV CPM thus mirrors the growing evidence that implicates the default mode network as being involved in high mind-wandering while simultaneously acknowledging that mind-wandering is an emergent construct that is likely associated with dynamic interactions across multiple canonical networks (Fox et al., 2015). Additionally, the default mode network and its various nodes have been critically implicated in the early pathophysiological processes of AD, with both the accumulation of *β*-amyloid plaques and tau tangles disproportionally aggregating in the densely connected midline structures of the posteromedial cortices and the medial prefrontal cortex (Buckner et al., 2005; Elman et al., 2016), and the medial temporal (Adams et al., 2019; Kaufman et al., 2018), respectively. Thus, our study, showing an association between the high RT_CV CPM that includes a large representation from the default mode network and AD pathophysiology, lends support to a potential link between mind-wandering and AD neurodegeneration.

It is also important to note, however, that the default mode network dysfunction lacks specificity, with default mode network alterations noted across a wide range of psychiatric (Whitfield-Gabrieli & Ford, 2012) and neurological disorders (Mohan et al., 2016). This network has also been implicated in cognitive processes beyond mind-wandering (e.g., social cognition; Buckner et al., 2008; Li et al., 2014; Mars et al., 2012). Additionally, even though the default mode network is central to mind-wandering and AD pathophysiology, there is also newer literature that questions the centrality of the default mode network in early AD pathophysiology (Buckley et al., 2017; Hahn et al., 2019; Pereira et al., 2021; Tahmi et al., 2020) and implicates the involvement of other large-scale brain systems. Notably, there is growing evidence from neuroimaging investigations (Groot et al., 2020; Wang, Beckmann, et al., 2018; Yamashita et al., 2021) and meta-analytic evidence (Fox et al., 2014) suggesting the involvement of other large-scale canonical networks, such as the fronto-parietal, dorsal attention, somatomotor, and salience networks, along with the functional coupling between these networks, in subserving mind-wandering (Groot et al., 2020). Additionally, the relationship between default mode network connectivity and AD is now recognized to be potentially less robust than previously indicated (Tahmi et al., 2020). Instead, it appears to be influenced by factors such as amyloid burden and specific cognitive submeasures (Buckley et al., 2017; Pereira et al., 2021).

To systematically examine the contribution of individual canonical networks, we elected to further explore the predictive contributions of the key networks via a computational lesion method. In selecting networks to be lesioned, we included the default mode network and the dorsal attention network due to their longstanding associations with mind-wandering (Christoff et al., 2016; Fox et al., 2015). Additionally, the functional connectivity of the fronto-parietal network has been posited as potentially critical to the shifts in mind-wandering behavior among older adults with dementia (O’Callaghan et al., 2019). Finally, the ventral attention network was included due to its overrepresentation in our RT_CV CPM. The computational lesioning of each of the four chosen networks provided evidence to the robustness of the whole-brain RT_CV CPM in support our initial hypothesis that a whole-brain neural marker of mind-wandering is associated with AD pathophysiology over and above that of individual canonical networks. Since the predictive power of the RT_CV CPM was retained at each of the computational lesioning, we argue that it is the combined connectivity patterns across the identified connectome that plays a role in that predictive utility, not merely that of the specific networks. Taken together, these findings lend credence to the hypothesized links between mind-wandering and AD pathophysiology.

Confirming the association between mind-wandering and cognitive performance (Mooneyham & Schooler, 2013), we found that network strength in the high RT_CV CPM was further associated with both global cognition and episodic memory. Of note, global cognition has been shown to consistently decline with age (Wilson et al., 2020) and with disease severity over time (Soldan et al., 2016). Indeed, global cognition measures, such as the PACC are sensitive to A*β*-related cognitive decline, and are frequently employed as a diagnostic screening tool for AD (Donohue et al., 2014). However, the relationship between mind-wandering and global cognition remains tangential, outside of domain-specific task performances (see Randall et al., 2014, for a review). In our study, extending prior work, we demonstrate that network strength in the functional connections predictive of high mind-wandering is further associated with lower global cognition in a large sample of older adults.

Furthermore, our results show that network strengths in the high RT_CV CPM are also strongly associated with poorer episodic memory. That is, stronger network functional connectivity for regions that predicted high response time variability is linked to poorer memory. Since memory declines are traditionally seen as the first casualty of AD-related neurodegeneration (Jahn, 2013) with prodromal memory deficits often being employed to indicate potential disease onset, the association of a neural model of mind-wandering with memory is notable. Additionally, mind-wandering has traditionally been closely tied to executive control—either as a function or a failure of it (Kane & McVay, 2012)—even though executive function itself is a broad term comprising multiple top-down cognitive processes (Miyake et al., 2000). While declines in these same processes have been demonstrated to be important tools in diagnosing AD (see Guarino et al., 2019, for a review), our results showing that executive function was not significantly associated with RT_CV network strengths could potentially point to the heterogenous nature of either mind-wandering, executive function, or both. Despite this, our results lend credence to the position that mind-wandering may be well positioned to be a potent biomarker for AD given the important ramifications that AD pathophysiology has on global cognition (Soldan et al., 2016) and memory (Jahn, 2013).

Though the current study demonstrated the association between a neural model of response time variability and AD pathophysiology, several limitations remain. Critically, we employed a neural signature of response time variability as an indirect marker of mind-wandering to examine its relationship with AD pathophysiology. Although there has been evidence for the use of behavioral variability as an indirect index of mind-wandering (Mrazek et al., 2012; Whitmoyer et al., 2020), there remains much debate as to the precise modality of the phenomenon. Within the literature, self-reported thought probes, other behavioral measures, and neurocognitive measures all represent potential markers of mind-wandering (Martinon et al., 2019; Smallwood & Schooler, 2015). As such, our findings represent only one aspect of mind-wandering, and future studies could explore the disparate aspects of mind-wandering that may be involved in Alzheimer’s disease pathophysiology.

Furthermore, the cross-sectional nature of our analyses provides only a snapshot of how a mind-wandering connectome might interact with AD pathophysiology at a single time point. Additionally, we note that model performance with AD pathophysiology associations, though statistically significant, remains weak (*ρ* = .137). While this is indeed lower than the predictive power seen in connectome-based modeling of other cognitive constructs (Avery et al., 2020; Barron et al., 2021; Finn et al., 2015; Lin et al., 2018; Manglani et al., 2022; Rosenberg et al., 2016), prior work has also shown that these associations tended to be lower when task-based CPMs are tested on resting-state scans (see Greene et al., 2018). In fact, in our original analyses of the RT_CV model (Gbadeyan et al., 2022), although the model derived on task was significant (*ρ* = .25), employing resting-state data to test the generalizability of the model in an independent dataset resulted in effects comparable to the ones observed in the current study (*ρ* = .15 for the combined model).

Finally, although previous studies have linked RT_CV with mind-wandering (Bastian & Sackur, 2013; Gbadeyan et al., 2022; Whitmoyer et al., 2020; Seli et al., 2013), trait-like variables, such as intelligence and *g*-factor, may also potentially underlie the associations observed between functionally relevant edges found in the RT_CV connectome and global cognition measures (Doebler & Scheffler, 2016). The ADNI dataset lacks measures of general intelligence (combining both fluid and crystallized intelligence) to tease apart this association. Nonetheless, research investigating the relationship between intelligence and RT_CV has suggested small effect sizes (*r*^2^ ≈ 4%–9%; Doebler & Scheffler, 2016) while studies examining correlations between RT_CV and other mind-wandering measures (e.g., sensitivity *d*′, probe-measured task unrelated thoughts, etc.) typically find larger effect sizes (*r*^2^ ≈ 9%–36%; see Kane & McVay, 2012). Thus, although we cannot completely rule out the possibility that intelligence underlies our findings, extent evidence suggests that mind-wandering nevertheless plays a significant role.

Despite the limitations, the current study is the first to successfully establish the novel associations between a behaviorally measured mind-wandering neural signature with cerebrospinal fluid pathophysiology and cognitive functioning in a large cohort of mixed pathology participants and healthy controls (*n* = 289). The robustness of our findings is further supported by continued significant associations following computationally lesioning of networks thought to be critical to mind-wandering. Altogether, our findings offer a glimpse at the neural underpinnings of mind-wandering and their possible links to healthy and diseased aging. Future work on different mind-wandering modalities may further shed light on this relationship and allow for a more comprehensive understanding of this relationship.

## ACKNOWLEDGMENTS

Data collection and sharing for this project was funded by the Alzheimer’s Disease Neuroimaging Initiative (ADNI) (National Institutes of Health Grant U01 AG024904) and DOD ADNI (Department of Defense award number W81XWH-12-2-0012). ADNI is funded by the National Institute on Aging, the National Institute of Biomedical Imaging and Bioengineering, and through generous contributions from the following: AbbVie, Alzheimer’s Association; Alzheimer’s Drug Discovery Foundation; Araclon Biotech; BioClinica, Inc.; Biogen; Bristol-Myers Squibb Company; CereSpir, Inc.; Cogstate; Eisai Inc.; Elan Pharmaceuticals, Inc.; Eli Lilly and Company; EuroImmun; F. Hoffmann-La Roche Ltd and its affiliated company Genentech, Inc.; Fujirebio; GE Healthcare; IXICO Ltd.; Janssen Alzheimer Immunotherapy Research & Development, LLC.; Johnson & Johnson Pharmaceutical Research & Development LLC.; Lumosity; Lundbeck; Merck & Co., Inc.; Meso Scale Diagnostics, LLC.; NeuroRx Research; Neurotrack Technologies; Novartis Pharmaceuticals Corporation; Pfizer Inc.; Piramal Imaging; Servier; Takeda Pharmaceutical Company; and Transition Therapeutics. The Canadian Institutes of Health Research is providing funds to support ADNI clinical sites in Canada. Private sector contributions are facilitated by the Foundation for the National Institutes of Health (www.fnih.org). The grantee organization is the Northern California Institute for Research and Education, and the study is coordinated by the Alzheimer’s Therapeutic Research Institute at the University of Southern California. ADNI data are disseminated by the Laboratory for Neuro Imaging at the University of Southern California.

## SUPPORTING INFORMATION

Supporting information for this article is available at https://doi.org/10.1162/netn_a_00373. Data used in preparation of this article were obtained from the Alzheimer’s Disease Neuroimaging Initiative (ADNI) database (adni.loni.usc.edu). As such, the investigators within the ADNI contributed to the design and implementation of ADNI and/or provided data but did not participate in analysis or writing of this report. A complete listing of ADNI investigators can be found at: https://adni.loni.usc.edu/wpcontent/uploads/how_to_apply/ADNI_Acknowledgement_List.pdf.

## AUTHOR CONTRIBUTIONS

James Teng: Formal analysis; Methodology; Writing – original draft; Writing – review & editing. Michael R. McKenna: Data curation; Methodology; Validation. Oyetunde Gbadeyan: Formal analysis; Validation; Writing – review & editing. Ruchika S. Prakash: Conceptualization; Funding acquisition; Supervision; Writing – review & editing.

## FUNDING INFORMATION

Ruchika S. Prakash, National Institute on Aging (https://dx.doi.org/10.13039/100000049), Award ID: R01AG054427.

## Supplementary Material



## TECHNICAL TERMS

p-tau: Phosphorylated tau (p-tau) are proteins that typically help maintain the stability of axonal microtubules in a healthy neuron. In Alzheimer’s disease, microtubules instability can lead to the aggregation of tau and subsequently the disease’s characteristic neurofibrillary tangles.
A*β*_42_: A*β*_42_ is a form of amyloid beta, an important component of amyloid plaques found in the brain of individuals with Alzheimer’s disease.
Global cognition: Global cognition measures an individual’s overall mental abilities across multiple domains.
Episodic memory: Episodic memory is a type of consciously recollected memory from personal experience, and involves the ability to learn, store, and retrieve such memories.
Executive functioning: Executive functioning is a set of cognitive processes that facilitate goal-directed behavior via focused attention, planning, behavior monitoring and selection.
Edges: Edges indicate a possible functional “connection” between two specific nodes in the brain.
