# Supplementary material for: Linking the neural signature of response time variability to Alzheimer’s disease pathology and cognitive functioning
Source: Netw Neurosci. 2024 Oct 1;8(3):697–713. doi: 10.1162/netn_a_00373 (PMC11340992; doi:10.1162/netn_a_00373)
Supplement: Supplementary file 1 [file netn-8-3-697-s001.pdf]

Supplementary Materials

Classification of Diagnostic Status in the ADNI Sample: Participants were classified into diagnostic categories using a battery of subjective reports, as well as neuropsychological and physician assessments. In particular, participants were categorized as having mild cognitive impairment (MCI) if they had: 1) an MMSE score ≥ 24, 2) scored within their education-adjusted range for the Logical Memory II subscale (EMCI: 9-11 for 16+ years of education, 5-9 for 8-15 years of education, 3-6 for 0-7 years of education; LMCI: ≤8 for 16+ of education, ≤4 for 8-15 years of education, ≤2 for 0-7 years of education), 3) a Clinical Dementia Rating score of 0.5, 4) self- or partner-reported memory complaint(s), and 5) reported absence of AD dementia or any other neurological conditions. Similarly, participants were classified as having AD if they had: 1) MMSE score between 20-26, 2) scored below their education-adjusted cut-offs for the Logical Memory Scale II (8 for 16+ of education, ≤4 for 8-15 years of education, ≤2 for 0-7 years of education), 3) had a Clinical Dementia Rating score of 0.5 or 1.0, and 4) self- or partner-reported memory complaints.

MRI Data Acquisition: All scans in the ADNI dataset were acquired on a 3-Tesla MRI scanners. Anatomical T1-weighted 3D magnetisation-prepared rapid gradient echo (MPRAGE) images for ADNIGO and ADNI2 (n=106) were acquired using the following parameters: TR = 6700 ms, TE = 3.1 ms, flip angle = 9°, field of view = 256 x 256 x 170 mm^3, slice thickness = 1.20 mm, voxel size standard = 1.0 mm^3, voxel size accelerated = 1.11 mm^3. MPRAGE images for ADNI3 scans (n=183) were acquired using the

following parameters: TR = 2300 ms, TE = min full echo, TI = 900 ms, 208 x 240 x 256 mm, voxel size = 1 mm^3.

Resting-state functional images were acquired using an echo-planar imaging (EPI) sequence using the following parameters for ADNIGO and ADNI2: TR = 3000 ms, TE = 30 ms, flip angle = 80°, field of view = 212 x 212 mm^2, slice thickness = 3.13 mm, voxel size = 3.125 mm^3, 48 sagittal slices. Of these participants, two participants had scans with a TR of 2250 ms, and one with a TR of 3090 ms, but this between-subject discrepancy was accounted for using participant-specific TR calculations for all subsequent preprocessing steps. ADNI3 participants used the following parameters for the resting-state sequence: TR = 3000 ms, TE = 30 ms, flip angle = 90°, field of view = 220 x 220 x 163 mm, slice thickness = 3.4 mm^3.

MRI Data Preprocessing: MRI data was organized and preprocessed in the standardized fMRIprep pipeline (v20.2.0.LTS; Esteban et al., 2019; pipeline details can be found at: https://fmriprep.readthedocs.io/en/latest/workflows.html). Briefly, anatomical preprocessing involved bias field correction using N4BiasFieldCorrection v2.1.0 (Tustison et al., 2010), skull stripping using antsBrainExtraction.sh v2.1.0, and nonlinear registration to MNI space with antsRegistration tool (Avants et al., 2008). Finally, the brain-extracted T1-weighted image was segmented into cerebrospinal fluid, white matter, and grey matter tissue using FAST (Zhang et al., 2001). Functional data was preprocessed as follows: 1) removing the first three volumes of the functional run to allow BOLD signal stabilization, 2) Susceptibility distortion correction using 3dQwarp (Cox and Hyde, 1997), 3) Boundary-based registration to corresponding T1-weighted images using with nine degrees-of-freedom (Greve and Fischl, 2009) with motion

corrected using FSL MCFLIRT v5.0.9 (Jenkinson et al., 2012), 4) Slice time correction using 3dTshift (Cox et al., 1996), 5) Motion correcting transformations, functional-to-T1w transformation, and T1w-to-MNI-template warp were concatenated and applied in a single step with antsApplyTransforms (ANTs v2.1.0) using Lanczos interpolation, and 6) Extraction of physiological noise and other nuisance regressors using the CompCor procedure (Behzadi et al., 2007), including framewise displacement and single timepoint regressor for motion outlier timeframes (framewise displacement value ≥ 0.5 mm), mean global signal (Power et al., 2014), white matter and cerebrospinal fluid signal, as well as six rigid body motion parameters and their temporal derivatives, together with their respective squares (Friston et al., 1996).

Following this, additional postprocessing was performed by: 1) denoising using the signal.clean function within Nilearn (Abraham et al., 2014), which allowed for the removal of the extracted nuisance regressors: mean white matter, cerebrospinal fluid, and global signal, 6 rigid body motion parameters and their temporal derivatives, together with their respective squares, and 2) high-pass filtering to remove signal frequencies of < 0.01 Hz. To account for additional motion-related confounds, we included a spike regressor for or each frame with framewise displacement value > 0.5 mm. As well, spatial smoothing was not performed as network analysis involved parcellation into a functional atlas (Alakörkkö et al., 2017).

References

Abraham, A., Pedregosa, F., Eickenberg, M., Gervais, P., Mueller, A., Kossaifi, J., ... & Varoquaux, G. (2014). Machine learning for neuroimaging with scikit-learn. *Frontiers in Neuroinformatics*, 14.

Alakörkkö, T., Saarimäki, H., Glerean, E., Saramäki, J., & Korhonen, O. (2017). Effects of spatial smoothing on functional brain networks. *European Journal of Neuroscience*, *46*(9), 2471-2480.

Avants, B. B., Epstein, C. L., Grossman, M., & Gee, J. C. (2008). Symmetric diffeomorphic image registration with cross-correlation: evaluating automated labeling of elderly and neurodegenerative brain. *Medical Image Analysis*, *12*(1), 26-41.

Behzadi, Y., Restom, K., Liau, J., & Liu, T. T. (2007). A component based noise correction method (CompCor) for BOLD and perfusion based fMRI. *NeuroImage*, *37*(1), 90-101.

Cox, R. W. (1996). AFNI: software for analysis and visualization of functional magnetic resonance neuroimages. *Computers and Biomedical Research*, *29*(3), 162-173.

Cox, R. W., & Hyde, J. S. (1997). Software tools for analysis and visualization of fMRI data. *NMR in Biomedicine: An International Journal Devoted to the Development and Application of Magnetic Resonance In Vivo*, *10*(4-5), 171-178.

Esteban, O., Markiewicz, C. J., Blair, R. W., Moodie, C. A., Isik, A. I., Erramuzpe, A., ... & Gorgolewski, K. J. (2019). fMRIPrep: a robust preprocessing pipeline for functional MRI. *Nature Methods*, *16*(1), 111-116.

Friston, K. J., Williams, S., Howard, R., Frackowiak, R. S., & Turner, R. (1996). Movement-related effects in fMRI time-series. *Magnetic Resonance in Medicine*, *35*(3), 346-355.

Gorgolewski, K. J., Auer, T., Calhoun, V. D., Craddock, R. C., Das, S., Duff, E. P., ... & Poldrack, R. A. (2016). The brain imaging data structure, a format for organizing and describing outputs of neuroimaging experiments. *Scientific Data*, *3*(1), 1-9.

Greve, D. N., & Fischl, B. (2009). Accurate and robust brain image alignment using boundary-based registration. *NeuroImage*, *48*(1), 63-72.

Jenkinson, M., Beckmann, C. F., Behrens, T. E., Woolrich, M. W., & Smith, S. M. (2012). FSL. *NeuroImage*, *62*(2), 782-790.

Power, J. D., Mitra, A., Laumann, T. O., Snyder, A. Z., Schlaggar, B. L., & Petersen, S. E. (2014). Methods to detect, characterize, and remove motion artifact in resting state fMRI. *NeuroImage*, *84*, 320-341.

Tustison, N. J., Avants, B. B., Cook, P. A., Zheng, Y., Egan, A., Yushkevich, P. A., & Gee, J. C. (2010). N4ITK: improved N3 bias correction. *IEEE Transactions on Medical Imaging*, *29*(6), 1310-1320.

Zhang, Y., Brady, M., & Smith, S. (2001). Segmentation of brain MR images through a hidden Markov random field model and the expectation-maximization algorithm. *IEEE Transactions on Medical Imaging*, *20*(1), 45-57.
